# Association between composite dietary antioxidant index and cognitive function impairment in the elderly: evidence from NHANES 2011–2014

**DOI:** 10.3389/fneur.2025.1529989

**Published:** 2025-04-25

**Authors:** Yingying Qian, Qiang Liu, Tianlang Li

**Affiliations:** Department of Geriatrics, Second Affiliated Hospital, School of Medicine, Zhejiang University, Hangzhou, China

**Keywords:** CDAI, cognitive function impairment, NHANES, cross-sectional study, nomogram model

## Abstract

**Objective:**

Antioxidant-rich diets are posited as protective factors against cognitive function impairment. The Composite Dietary Antioxidant Index (CDAI) serves as a pivotal measure of antioxidant intake, yet its relationship with cognitive function impairment has been sparsely investigated. Herein, the purpose of this study was to investigate the relationship between CDAI and cognitive function impairment.

**Methods:**

An analysis of data from the National Health and Nutrition Examination Survey (NHANES) spanning 2011 to 2014 was conducted to examine the relationship between the CDAI and cognitive function impairment by multivariate logistic regression, and its nonlinearity was verified by restricted cubic spline (RCS) regression. Moreover, a risk prediction nomogram model containing the key factors determined by logistic regression methods was constructed to estimate the probability of cognitive function impairment in older adults.

**Results:**

Compared with participants with normal cognitive performance, those with low cognitive performance were likely to have higher age, lower education, lower household income, and lower CDAI score. In a multivariate logistic regression model adjusted for confounding variables, the CDAI score was associated with the CERAD word learning subtest was still significant, the adjusted odds ratios (ORs) with 95% confidence intervals (CIs) was 0.94 (0.90,0.98), while the association with AFT and DSST was not statistically significant. The RCS curves indicate that there was a smooth L-shaped correlation between CDAI index and cognitive performance. Moreover, the nomogram model based on the key factors determined by logistic regression has a good predictive value for cognitive function impairment (AUC = 0.747, 95%CI:0.726–0.768).

**Conclusion:**

Our study determined a nonlinear and negative association between CDAI and cognitive function impairment in the US elderly population. And a risk prediction nomogram model was constructed to estimate the probability of cognitive function impairment in older adults.

## 1 Introduction

With the advent of global aging, the rising incidence of cognitive function impairment and dementia will become a major public health concern for humanity (1). Projections suggest that by 2050, estimated 152 million people worldwide will be living with cognitive function impairment (2). The disease severely affects the quality of life for patients, as well as imposing a heavy socio-economic burden.

Given the irreversible nature, exploring modifiable lifestyle and risk factors is essential in the endeavor to delay and prevent cognitive function impairment (3). Diet stands out as a modifiable determinant worthy of investigation. Studies indicate that adherence to a higher-quality diet, consistent with the Dietary Guidelines for Americans (DGA), is related to better cognitive performance among the aging population in the United States (4).

Despite extensive research into cognitive function impairment, its etiology, pathogenesis and treatment remain incompletely understood. Previous investigations have suggested that inflammation, oxidative stress, metabolic abnormalities may be involved in the development of cognitive function impairment (5, 6). The brain is particularly vulnerable to oxidative stress due to its distinctive structure, such as its high oxygen utilization, rich unsaturated fatty acid composition, and relatively low antioxidant capacity (7). Research indicates that regular consumption of antioxidants in the diet can enhance antioxidant defense mechanisms and mitigate oxidative stress by elevating plasma antioxidant concentrations. Studies have shown that daily dietary consumption of antioxidants can enhance antioxidant defense and weaken oxidative stress elevating antioxidant level in the blood (8). Modifying dietary patterns may thus serve as an effective strategy to alleviate cognitive function impairment by reducing systemic oxidative stress levels.

The Composite Dietary Antioxidant Index (CDAI) is an effective and reliable nutritional tool for assessing the overall antioxidant properties of an individual’s diet (9). It is a composite score summarizing multiple dietary antioxidants, including vitamins A, C, and E, carotenoids, selenium, and zinc (10, 11). CDAI is a composite index, and previous studies have generally been limited to examining the relationship between specific antioxidants and cognitive performance, but there has been limited research on the potential combined benefits of antioxidants on cognitive impairment. Therefore, we analyzed data from the National Health and Nutrition Examination Survey (NHANES) to investigate potential correlations between the CDAI and cognitive function impairment, with the goal of decreasing the incidence of cognitive function impairment through dietary interventions.

## 2 Data and methods

### 2.1 Study population

**Figure 1:**
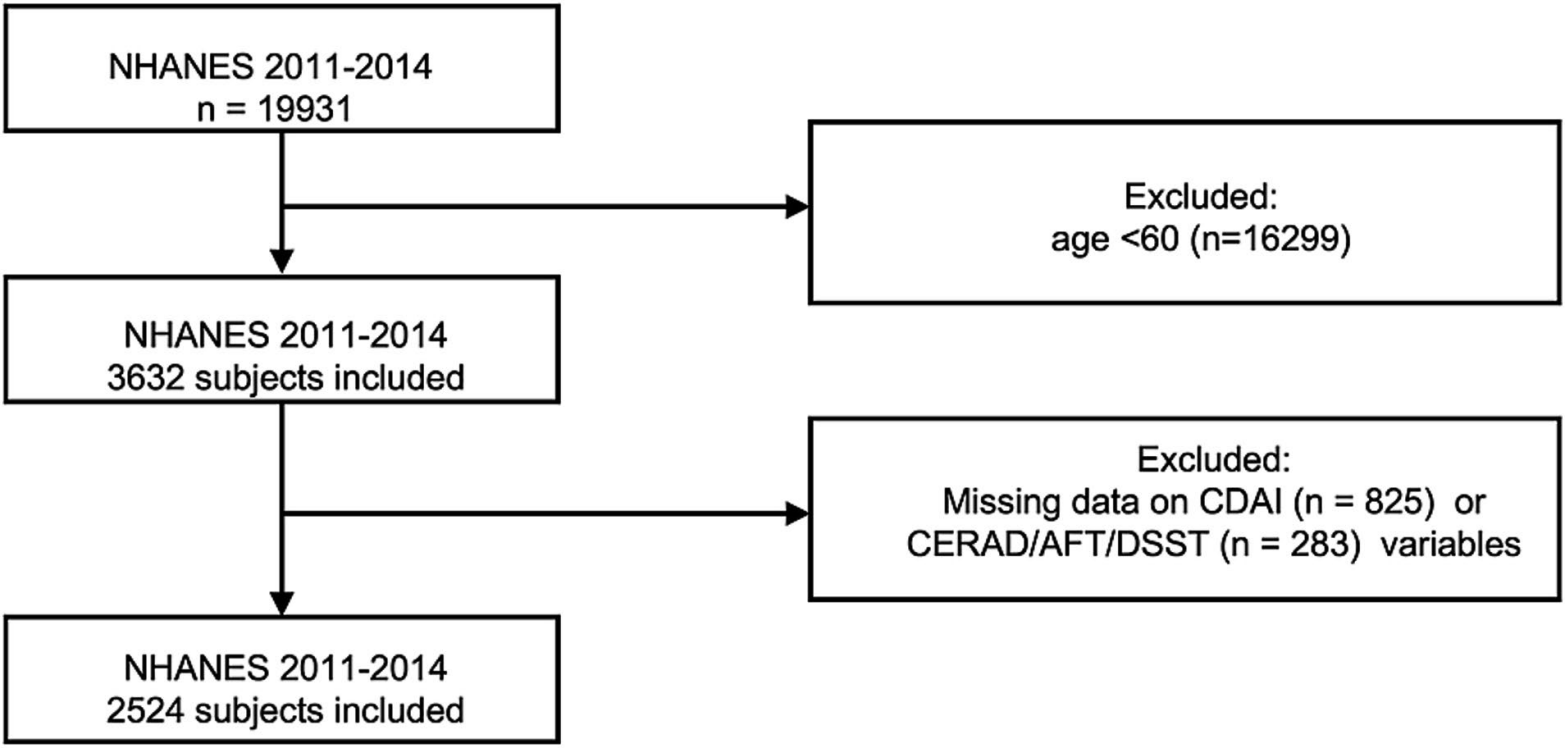
Flowchart illustrating the sample selection process from NHANES 2011–2014.

The NHANES is a large cross-sectional survey conducted by the U.S. National Centers for Disease Control (CDC). This survey was approved by the Ethics Review Board of the National Center for Health Statistics (NCHS), and all participants provided written consent. In this study, we selected two cycles, specifically NHANES 2011–2012 and 2013–2014, as these cognitive tests were performed specifically during the two cycles. From 2011 to 2014, a total of 19,931 individuals participated in NHANES. Inclusion Criteria: Participants aged ≥ 60 years. Exclusion Criteria: (1) participants with missing dietary intake data for key antioxidants, including carotenoids, manganese, selenium, and vitamins A, C, and E; (2) participants with incomplete cognitive test data. Multiple interpolation is used to interpolate the missing covariates (Supplementary Table S1) (12). After applying these rigorous exclusion criteria, a total of 2,524 eligible elderly adults were included in the final analysis (Figure 1).

### 2.2 Dietary assessment and CDAI calculation

Dietary intake data among NHANES participants were collected through 24-h dietary recall interviews conducted on 2 days. The first diet recall was done in person, and the follow-up recall was conducted by phone 3–10 days later. The average daily intake was calculated based on the dietary recalls from both days. The calculation of CDAI for each subject followed the methodology which involved six essential nutrients (carotenoids, selenium, zinc, vitamins A, C, and E) obtained from dietary sources (13). The CDAI was determined by subtracting the mean value and dividing the outcome by the standard deviation for each of the six dietary minerals and vitamins, as outlined below:


$$\mathrm{CDAI}=\sum_{i=1}^{n=6}\frac{\mathrm{IndividualIntake}-\mathrm{Mean}}{SD}$$

### 2.3 Cognitive assessment

Cognitive function in the 2011–2014 NHANES cohort was assessed by three tests, including the Consortium of Alzheimer’s Word Learning Registers (CERAD), the Animal Fluency Test (AFT), and the Digit Symbol Substitution Test (DSST).

The CERAD (14) consists of three consecutive learning tests and a delayed recall test to assess both immediate and delayed memory. During the learning phase, participants were presented with 10 unrelated words sequentially, either visually or audibly, on a monitor and then prompted to recall as many words as possible immediately. After three rounds of learning tests, the cumulative score for immediate learning and recall was computed by tallying the number of correct responses across all rounds for each participant. Delayed recall trials commenced approximately 8–10 min after the initial immediate recall tests. Each test was scored on a scale from 0 to 10, with the final score for the CERAD test being the sum of the three consecutive learning trials and the delayed recall trial.

The AFT (15) is employed to evaluate categorical verbal fluency, which is an essential component of executive function. Participants are instructed to verbally list as many different animals as possible within a time limit of 1 min, and each correctly named animal earning one point. Total AFT score is then calculated by summing the count of correctly identified animals.

The DSST (16) is utilized to evaluate processing velocity, sustained attention, and executive memory. Participants are presented with a paper form featuring a key at the top, associating numbers with nine symbols. Their task is to match these symbols to corresponding numbers within 133 boxes, all within a time frame of 2 min. The final DSST score is determined by tallying the number of correctly matched pairs.

Now no definitive gold standard threshold exists for the CERAD, AFT and DSST tests, the report of NCHS has recommended using the 25th percentile as a cutoff for analyzing low cognitive performers (17). Therefore, in this study, the 25th percentile was utilized as the benchmark for identifying cognitive function impairment. Participants meeting the criteria of 21 scores in CERAD, 13 scores in AFT, and 33 scores in DSST were classified as exhibiting impaired cognitive function, which is also consistent with previous studies (18–22).

### 2.4 Covariates

To comprehensively analyze the relationship between CDAI and cognitive function impairment, this study included various covariates such as demographic characteristics, lifestyle factors, and health status, which have been shown to be associated with cognitive function according to previous literature. Demographic characteristics include age, sex, race, poverty income ratio (PIR), and education level. Lifestyle variables include smoking, alcohol consumption and physical activity. More than 100 cigarettes smoked in a person’s lifetime are considered smoking. The Global Physical Activity Questionnaire was used to calculate metabolic equivalent (MET) to assess physical activity, with MET values of less than 600 min per week defined as inactivity. Health status variables included chronic kidney disease (CKD), high blood pressure, depression and diabetes, as determined by physician diagnostic notes or self-reports. More details can be found in the NHANES Laboratory Procedures Manual (23).

### 2.5 Statistical analysis

In this study, continuous variables were presented as means ± standard error (mean ± SE), and categories were presented as proportions. Participants were divided into cognitive normal and cognitive function impairment groups, and descriptive analysis was conducted between these groups.

Univariate and multivariate logistic regression analyses were performed to explore the relationship between CDAI scores and cognitive function. No covariates were adjusted in Model 1. Model 2 was adjusted for age, gender and race. Model 3 was further adjusted for educational attainment, marital status, PIR, smoke, alcohol, BMI, stroke, CKD, diabetes, hypertension, depression, physical activity and intake of calories.

Furthermore, the possible dose–response association between CDAI and cognitive performances was examined using Restricted Cubic Spline (RCS) curves, which offer a flexible method for modeling and visualizing potential non-linear associations between variables without requiring *a priori* assumptions about the functional form of the relationship. Moreover, subgroup analyses based on age, gender, BMI, marital status, education level and race were conducted to further investigate the association between CDAI scores and cognitive function.

A nomogram is a graphical tool derived from predictive models, allowing users to estimate the probability of an outcome by integrating multiple variables. It provides an effective way to communicate multifactorial predictions in a straightforward and accessible manner. In our study, the nomogram was constructed based on multivariable logistic regression models to estimate the likelihood of cognitive impairment associated with the CDAI. Model performance was assessed by using the ROC curve. Statistical analyses were performed using R 4.3.1 software, utilizing MEC weights. *p* < 0.05 (two-tailed) was considered statistically significant.

## 3 Results

### 3.1 Characteristics of the study population

Table 1 shows the baseline characteristics of all eligible participants. Data from a total of 2,524 participants from NHANES was included in this retrospective study. Demographic information, including age, gender, race, marital status, education level, household income, BMI, drinking and smoking habits, was categorized accordingly.

**Table 1:** Baseline characteristics of the study population from NHANES2011-2014.

Characteristic	CERAD			AFT			DSST		
	Normal (*N* = 1,818)	Low (*N* = 706)	*p*	Normal (*N* = 1,794)	Low (*N* = 730)	*p*	Normal (*N* = 1,932)	Low (*N* = 592)	*p*
Age	66 (63,72)	73 (68,80)	<0.001	67 (63,73)	72 (65,80)	<0.001	67 (63,73)	75 (68,80)	<0.001
Sex			<0.001			0.809			0.857
Female	1,032 (55.9%)	275 (44.1%)		925 (53.1%)	382 (54.1%)		1,042 (53.2%)	265 (53.8%)	
Male	786 (44.1%)	431 (55.9%)		869 (46.9%)	348 (45.9%)		890 (46.8%)	327 (46.2%)	
Race			<0.001			<0.001			<0.001
Mexican American	136 (2.8%)	75 (5.4%)		155 (3.2%)	56 (4.3%)		135 (2.5%)	76 (9.1%)	
Non-Hispanic Black	426 (8.4%)	168 (9.5%)		348 (6.5%)	246 (16.3%)		387 (6.5%)	207 (22.4%)	
Non-Hispanic White	939 (80.5%)	330 (73.4%)		1,007 (82.9%)	262 (64.3%)		1,090 (83.1%)	179 (51.5%)	
Other Hispanic	157 (3.1%)	87 (6.9%)		160 (3.3%)	84 (6.3%)		136 (2.6%)	108 (13.3%)	
Other	160 (5.2%)	46 (4.8%)		124 (4.1%)	82 (8.8%)		184 (5.3%)	22 (3.7%)	
Marital status			<0.001			0.032			<0.001
Divorced	285 (13.0%)	72 (9.7%)		270 (12.4%)	87 (11.6%)		287 (12.2%)	70 (12.1%)	
Married	1,029 (65.5%)	384 (58.3%)		1,023 (65.5%)	390 (58.3%)		1,132 (66.4%)	281 (47.8%)	
Other*	459 (20.6%)	229 (30.9%)		459 (21.2%)	229 (28.6%)		480 (20.7%)	208 (36.6%)	
Separated	45 (0.9%)	21 (1.1%)		42 (0.9%)	24 (1.5%)		33 (0.6%)	33 (3.4%)	
Educational level			<0.001			<0.001			<0.001
High school or above	1,493 (88.8%)	427 (71.7%)		1,460 (88.2%)	460 (73.4%)		1,657 (89.9%)	263 (52.5%)	
Less than high school	325 (11.2%)	279 (28.3%)		334 (11.8%)	270 (26.6%)		275 (10.1%)	329 (47.5%)	
PIR	3.43 (1.88,5.00)	2.04 (1.19,3.97)	<0.001	3.43 (1.84,5.00)	2.23 (1.21,3.81)	<0.001	3.44 (1.92,5.00)	1.59 (0.98,2.31)	<0.001
BMI	28.20 (24.70,32.90)	27.20 (24.30,31.00)	0.023	28.00 (24.60,32.50)	27.70 (24.40,32.30)	0.749	28.00 (24.60,32.30)	27.80 (24.60,33.20)	0.818
Smoke			0.52			0.448			0.764
No	898 (50.6%)	349 (52.3%)		876 (50.4%)	371 (52.8%)		957 (51.1%)	290 (50.1%)	
Yes	920 (49.4%)	357 (47.7%)		918 (49.6%)	359 (47.2%)		975 (48.9%)	302 (49.9%)	
Alcohol			<0.001			<0.001			<0.001
No	557 (25.4%)	226 (32.6%)		514 (24.0%)	269 (38.0%)		559 (24.8%)	224 (41.1%)	
Yes	1,261 (74.6%)	480 (67.4%)		1,280 (76.0%)	461 (62.0%)		1,373 (75.2%)	368 (58.9%)	
Stroke			0.091			0.034			<0.001
No	1,717 (94.63)	638 (91.98)		1,696 (94.87)	659 (91.02)		1,838 (95.27)	517 (85.94)	
Yes	101 (5.37)	68 (8.02)		98 (5.13)	71 (8.98)		94 (4.73)	75 (14.06)	
CKD			<0.001			<0.001			<0.001
No	1,263 (73.18)	377 (54.02)		1,227 (71.86)	413 (58.18)		1,339 (72.65)	301 (44.28)	
Yes	555 (26.82)	329 (45.98)		567 (28.14)	317 (41.82)		593 (27.35)	291 (55.72)	
Diabetes			0.027			0.058			<0.001
No	1,062 (64.59)	369 (59.52)		1,055 (64.88)	376 (58.27)		1,160 (65.84)	271 (47.76)	
Yes	756 (35.41)	337 (40.48)		739 (35.12)	354 (41.73)		772 (34.16)	321 (52.24)	
Hypertension			<0.001			<0.001			<0.001
No	560 (36.19)	178 (21.69)		577 (35.85)	161 (22.43)		616 (35.11)	122 (18.82)	
Yes	1,258 (63.81)	528 (78.31)		1,217 (64.15)	569 (77.57)		1,316 (64.89)	470 (81.18)	
Depression			0.337			0.020			<0.001
No	1,672 (93.09)	630 (91.52)		1,663 (93.91)	639 (88.48)		1,795 (93.77)	507 (85.96)	
Yes	146 (6.91)	76 (8.48)		131 (6.09)	91 (11.52)		137 (6.23)	85 (14.04)	
Physical activity			0.132			0.596			<0.001
No	1,341 (75.65)	495 (71.48)		1,333 (75.05)	503 (73.51)		1,442 (76.73)	394 (61.44)	
Yes	477 (24.35)	211 (28.52)		461 (24.95)	227 (26.49)		490 (23.27)	198 (38.56)	
CDAI	0.53 (−1.69,3.01)	−0.55 (−2.89,1.38)	<0.001	0.56 (−1.66,2.82)	−0.91 (−2.96,1.81)	<0.001	0.48 (−1.69,2.82)	−1.43 (−3.31,1.01)	<0.001
Vitamin A	592.00 (394.50,871.50)	553.50 (355.50,842.00)	0.103	595.50 (397.50,854.00)	498.50 (337.50,880.50)	0.113	597.50 (397.50,879.50)	469.50 (287.00,759.50)	<0.001
Vitamin C	69.65 (34.65,119.15)	65.30 (31.40,110.35)	0.377	69.75 (34.25,118.45)	66.75 (31.75,109.15)	0.33	69.45 (34.20,119.05)	64.70 (31.40,107.35)	0.155
Vitamin E	8.02 (5.73,11.03)	6.20 (4.25, 8.93)	<0.001	8.05 (5.67,10.94)	6.28 (4.46, 8.91)	<0.001	7.87 (5.58,10.89)	5.87 (4.06, 8.48)	<0.001
Zinc	9.84 (7.42,13.00)	9.15 (6.85,12.22)	0.019	9.92 (7.49,12.97)	9.00 (6.37,11.80)	0.003	9.84 (7.45,12.95)	8.61 (5.82,11.69)	<0.001
Selenium	101.50 (76.00,130.50)	95.80 (72.05,119.50)	<0.001	102.75 (78.10,130.50)	89.10 (66.05,114.80)	<0.001	101.80 (77.50,129.20)	84.45 (63.60,115.40)	<0.001
Carotenoid	7038.50 (3672.50,14222.00)	5444.00 (2208.50,10611.50)	<0.001	7038.50 (3707.00,14115.00)	4945.50 (2051.50, 9988.00)	<0.001	7038.50 (3580.00,13726.50)	4487.50 (2212.50, 9489.00)	<0.001
Day1 intake energy	1,819 (1,387,2,355)	1,793 (1,267,2,400)	0.197	1,865 (1,431,2,413)	1,629 (1,229,2,276)	<0.001	1,855 (1,417,2,414)	1,519 (1,175,2032)	<0.001
Day2 intake energy	1,826 (1,403,2,353)	1,660 (1,191,2,138)	0.003	1,835 (1,419,2,349)	1,646 (1,161,2050)	<0.001	1,827 (1,405,2,336)	1,511 (1,105,1965)	<0.001
CERAD total scores	28 (25,32)	18 (15,20)	<0.001	28 (23,32)	22 (18,27)	<0.001	28 (23,32)	20 (17,25)	<0.001
AFT total scores	19 (15,22)	14 (11,17)	<0.001	19 (17,23)	11 (10,13)	<0.001	19 (15,22)	13 (10,16)	<0.001
DSST total scores	57 (46,66)	40 (30,49)	<0.001	56 (45,66)	40 (29,52)	<0.001	56 (46,65)	27 (21,31)	<0.001

Data are represented as the weighted proportion (%) or mean ± SD. *p* < 0.05 indicates statistical significance. Low cognitive performance was defined by CERAD total scores ≤ 21; low cognitive performance was defined by AFT total scores ≤ 13; low cognitive performance was defined by DSST total scores ≤ 33; *Represents widowed, never married and living with partner. PIR, Poverty-income ratio; BMI, Body mass index; CKD, chronic kidney disease; CERAD, Consortium to Establish a Registry for Alzheimer’s Disease; AFT, Animal Fluency Test; DSST, Digit Symbol Substitution Test.

In the CERAD word learning subtest, the CDAI of normal cognition and low cognition were 0.53 (−1.69,3.01) and −0.55 (−2.89,1.38), respectively. There were significant differences between the two groups in age, gender, race, marital status, education level, household income, alcohol consumption, and the presence of hypertension and CKD. However, no significant differences were observed in smoking habits, physical activity, BMI, or the presence of stroke, diabetes and depression. In AFT test, the CDAI of normal cognition and low cognition were 0.56 (−1.66,2.82) and −0.91 (−2.96,1.81), respectively. There were significant differences between the two groups in age, race, education level, family income, alcohol consumption and the presence of hypertension and CKD, but no significant differences in gender, marital status, smoking, physical activity, BMI or the presence of stroke, diabetes and depression. In the DSST test, the CDAI of normal cognition and low cognition were 0.48 (−1.69,2.82) and −1.43 (−3.31,1.01), respectively. There were significant differences between the two groups in age, race, marital status, education level, family income, alcohol consumption, physical activity and the presence of hypertension, CKD, stroke, and depression, but no significant differences in gender, smoking and BMI.

Overall, participants with low cognitive performance, compared to those with normal cognitive performance, exhibited higher age, lower educational attainment, lower household income, were more frequently accompanied by CKD and hypertension, and had lower CDAI scores (refer to Table 1 for details).

### 3.2 Association of CDAI with cognitive performance

**Figure 2:**
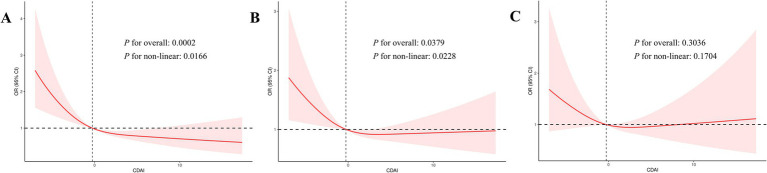
The RCS curves showing the association between CDAI and cognitive performance. **(A)** Association between CDAI and CERAD score. **(B)** Association between CDAI and AFT score. **(C)** Association between CDAI and DSST score.

Table 2 shows the association between CDAI score and cognitive performance, with a significant positive correlation between the two. Three models were established in this study. After adjusting factors such as age, gender and race (Model 2), linear regression analysis showed that the OR values of CDAI and CERAD scores, AFT scores and DSST scores were 0.92 (0.88,0.96), respectively. 0.93 (0.88,0.97) and 0.90 (0.86,0.95) were statistically significant. After fully adjusting for factors such as age, sex, race, educational attainment, marital status, PIR, smoke, alcohol, BMI, stroke, CKD, diabetes, hypertension, depression, physical activity and intake of calories (Model 3), CDAI and CERAD scores OR were 0.94 (0.90,0.98), which still had obvious significance (*p* = 0.014). However, in multiple logistic regression analysis, there was no statistically significant association between AFT test scores and DSST scores and CDAI scores (see Supplementary Table S2). In addition, we analyzed whether there is a nonlinear correlation between CDAI and cognition. The RCS curves indicate that there may be an L-shaped smooth correlation between CDAI index and cognitive test score, P-Nonlinear < 0.05 (Figure 2).

**Table 2:** The association between CADI score and cognitive performance.

Outcomes	Model 1		Model 2		Model 3	
	OR (95% CI)	*p*	OR (95% CI)	*p*	OR (95% CI)	*p*
CERAD	0.92 (0.88,0.95)	**<0.001**	0.92 (0.88,0.96)	**<0.001**	0.94 (0.90,0.98)	**0.014**
AFT	0.92 (0.87,0.96)	**0.001**	0.93 (0.88,0.97)	**0.003**	0.97 (0.92,1.02)	0.389
DSST	0.88 (0.84,0.93)	**<0.001**	0.90 (0.86,0.95)	**<0.001**	0.98 (0.93,1.02)	0.462

Low cognitive performance was defined by CERAD total scores ≤ 21; low cognitive performance was defined by AFT total scores ≤ 13; low cognitive performance was defined by DSST total scores ≤ 33. Model 1 lacked adjustments for covariates. Model 2 adjusted for age, sex, and race. Model 3 adjusted for age, sex, race, educational attainment, marital status, PIR, smoke, alcohol, BMI, day1 intake energy, and day2 intake energy, stroke, CKD, diabetes, hypertension, depression, physical activity. Bold values indicate statistical significance (*p* < 0.05).

**Figure 3:**
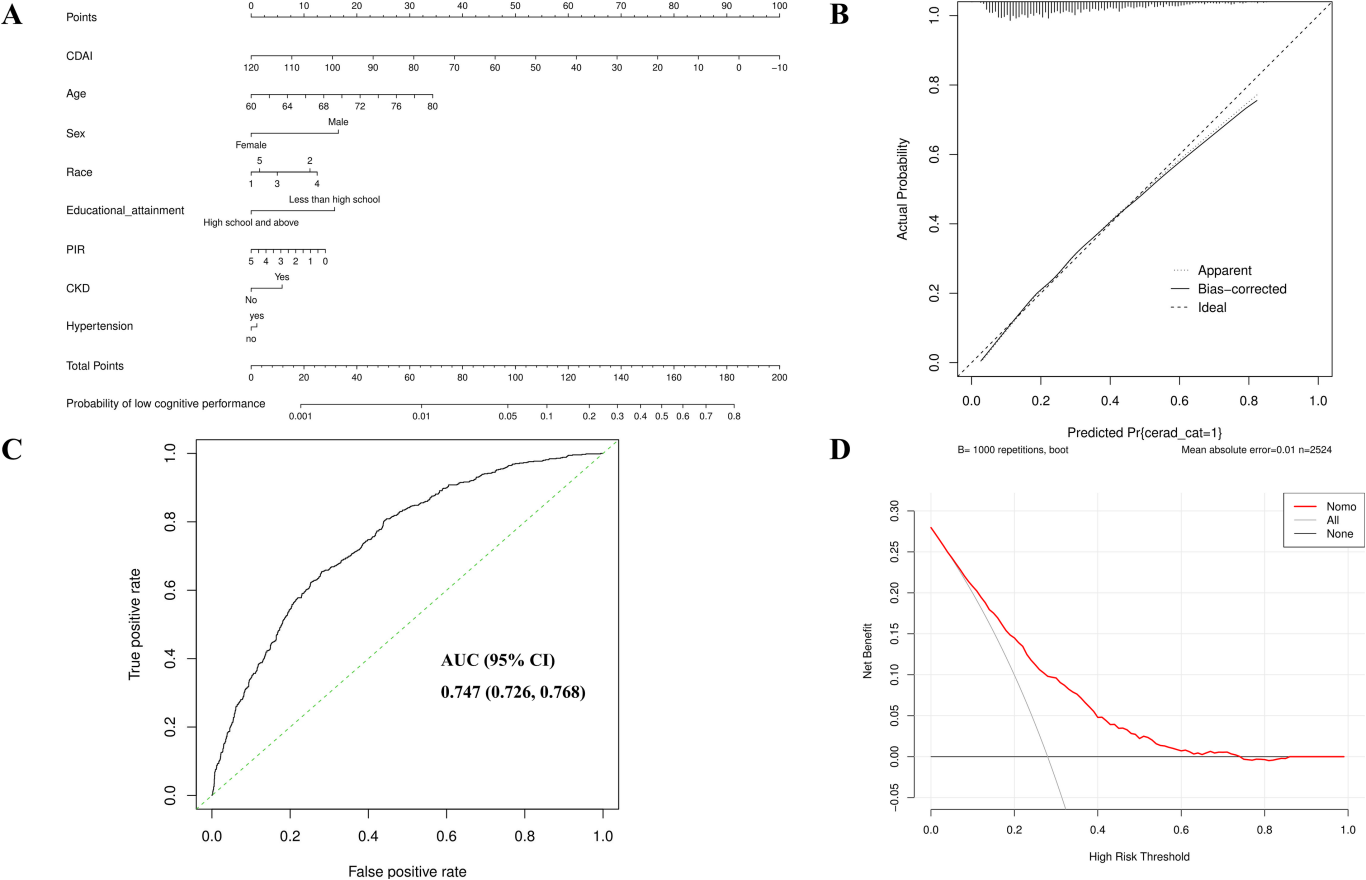
Development and validation of a nomogram model for predicting the risk of low cognitive performance. **(A)** A nomogram model based on CDAI, age, sex, race, educational attainment and PIR. Race categories are coded as follows: 1 = Non-Hispanic White; 2 = Mexican American; 3 = Non-Hispanic Black; 4 = Other Hispanic; 5 = Other Race (including multiracial individuals). **(B)** ROC curve evaluating the predictive power of the nomogram model. **(C)** Calibration curve assessing agreement between predicted and observed probabilities. **(D)** Decision curve analysis (DCA) demonstrating the clinical validity of the nomogram model.

Subgroup analyses were conducted to investigate the potential link between CDAI scores and risk of cognitive function impairment in different populations based on age, gender, race, marital status, educational level, PIR and BMI. As shown in Figure 3, more significant effects were observed in women, high school education and above, married or non-Hispanic White with CDAI scores, suggesting that these subgroups may benefit more from higher dietary antioxidant intake. The differences between subgroups were mostly insignificant, suggesting that the benefits of CDAI were consistent across populations.

### 3.3 A nomogram model for cognitive function impairment

Statistically significant variables from multiple logistic regression analysis were used to construct a predictive model of cognitive impairment risk, and Nomogram was used for visualization. As shown in Figure 3, each patient has its own score for each of the eight independent related factors (CDAI, Age, Gender, Race, Educational attainment, PIR, CKD and hypertension), all of which add up to a total score. According to the total score, the probability of low cognitive performance of each patient can be intuitively and conveniently obtained, which can be better used in clinical practice. The Nomogram model had a considerable predictive performance for Cognitive function impairment, which was validated by ROC curve (AUC = 0.747, 95%CI:0.726–0.768), and the sensitivity and specificity were 0.654 and 0.719, respectively (Table 3).

**Table 3:** Stratified analyses by age, gender, BMI, marital status, education level, and race.

Variables	CERAD		AFT		DSST	
	OR (95%CI)	*p*	OR (95%CI)	*p*	OR (95%CI)	*p*
Age, years						
60—64	0.934 (0.810, 1.077)	0.311	0.976 (0.869, 1.096)	0.654	1.006 (0.972, 1.042)	0.692
≥65	0.956 (0.915,0.999)	**0.046**	0.990 (0.932,1.052)	0.730	0.991 (0.912,1.076)	0.804
Gender						
Female	0.908 (0.856,0.963)	**0.004**	0.992 (0.915,1.075)	0.823	0.976 (0.892,1.068)	0.559
Male	0.959 (0.882,1.044)	0.300	0.945 (0.849, 1.051)	0.261	0.979 (0.909,1.054)	0.535
BMI, kg/m^2^						
≤25	0.892 (0.804,0.989)	**0.033**	0.994 (0.935,1.057)	0.833	0.991 (0.923, 1.065)	0.788
>25 and≤30	0.950 (0.833,1.083)	0.403	0.959 (0.885,1.038)	0.266	0.953 (0.843,1.077)	0.397
>30	0.965 (0.874,1.065)	0.436	0.961 (0.873,1.059)	0.383	0.997 (0.877, 1.132)	0.954
Educational attainment						
High school and above	0.930 (0.885,0.976)	**0.008**	0.965 (0.908, 1.025)	0.215	0.925 (0.864, 0.991)	**0.031**
Less than high school	0.992 (0.911,1.081)	0.844	1.056 (0.945,1.179)	0.298	1.139 (1.022, 1.270)	**0.024**
Marital status						
Married	0.909 (0.851,0.972)	**0.009**	0.973 (0.902, 1.049)	0.440	0.974 (0.890,1.066)	0.533
Divorced	0.977 (0.884,1.080)	0.626	0.957 (0.835, 1.097)	0.498	1.032 (0.887, 1.200)	0.658
Separated	0.985 (0.745, 1.304)	0.910	3.986 (1.285, 12.364)	**0.021**	1.185 (0.781, 1.796)	0.389
Other^*^	0.958 (0.902,1.017)	0.140	0.960 (0.871,1.058)	0.370	0.969 (0.860, 1.093)	0.581
Race						
Mexican American	0.870 (0.702, 1.079)	0.183	0.864 (0.729, 1.024)	0.085	0.902 (0.778, 1.046)	0.156
Non-Hispanic Black	1.022 (0.931, 1.123)	0.594	0.992 (0.900,1.093)	0.849	1.008 (0.910,1.117)	0.858
Non-Hispanic White	0.916 (0.868,0.967)	**0.004**	0.946 (0.884, 1.012)	0.098	0.932 (0.847, 1.025)	0.133
Other Hispanic	1.089 (0.948, 1.250)	0.423	1.137 (0.994, 1.301)	0.296	1.231 (1.055, 1.436)	0.217
Other Race—including multi-racial	0.921 (0.748, 1.135)	0.357	1.107 (0.950, 1.289)	0.148	0.998 (0.810, 1.229)	0.980

^*^Represents widowed, never married, and living with partner. Bold values indicate statistical significance (*p* < 0.05).

## 4 Discussion

Aging is a physiological process accompanied by the decline of physical and cognitive functions, the decline of cognitive function affects memory, attention, spatial learning, executive function, and may eventually progress to cognitive dysfunction or even dementia (24). Cognitive function impairment is common in the aging process. Understanding the connection between diet and cognitive abilities is critical for the prevention and management of cognitive function impairment (25). This cross-sectional study examined the association between CDAI and cognitive impairment in older adults in the United States. We observed a significant positive association between higher CDAI scores and cognitive function, suggesting that CDAI is a protective factor against the development of cognitive impairment and that antioxidant diets may reduce the risk of cognitive impairment. In multivariate logistic regression models adjusted for confounding variables, the relationship between higher CDAI scores and cognitive function CERAD test results was robust. Further, RCS curves showed an L-shaped nonlinear correlation between the CDAI index and cognitive performance.

Here, the results of the three different cognitive function tests were slightly different. After adjusting various covariates, the correlation between CDAI score and CERAD word learning subtest was still significant, while the correlation between CDAI score and AFT and DSST was not statistically significant, indicating that CDAI may have a significant effect on improving memory ability, but not in processing speed, sustained attention and executive function. Episodic memory impairment is a hallmark of dementia and serves as a critical diagnostic criterion for typical Alzheimer’s disease. Early detection of memory function impairment is pivotal for identifying high-risk populations at a preclinical stage of dementia. Furthermore, timely and proactive interventions targeting memory function can effectively enhance cognitive outcomes in elderly individuals. The CERAD Word Learning Subtest and the Word Delayed Recall Test comprehensively assess both immediate and delayed recall abilities, providing a measure of memory performance. CERAD scores are widely recognized as a reliable reflection of memory function. In our study, the observed correlation between the CDAI and CERAD outcomes was notably significant. This finding underscores the unique relevance and precision of CDAI as an indicator. It highlights its potential value in identifying dietary patterns associated with memory function and cognitive health.

Subgroup analyses revealed that more significant effects were observed in women, high school education and above, married or non-Hispanic White individuals with higher CDAI scores, suggesting that these subgroups may derive greater benefits from higher dietary antioxidant intake. Gender differences may be due to hormonal and metabolic pathways. Individuals with higher education levels likely possess greater health literacy. This enables them to make more informed dietary choices aligned with the principles of the CDAI. Additionally, marriage often provides a support system that encourages healthier lifestyle choices, including diet. Married individuals may benefit from shared meal preparation, which increases the likelihood of consuming balanced and nutritious meals. For non-Hispanic White individuals, cultural dietary habits and genetic differences in antioxidant metabolism may explain their greater benefit from higher CDAI scores.

In recent years, the relationship between cognitive performance and dietary patterns has garnered significant attention (26). Nutrients from food can influence immune responses and modulate neuroinflammatory processes involved in the pathogenesis of dementia and cognitive impairment (27). Specific dietary patterns, such as the Mediterranean and MIND diets, emphasize antioxidant-rich foods and have been linked to slower cognitive decline and reduced risk of Alzheimer’s disease. These patterns support the notion that long-term antioxidant intake contributes to cognitive resilience and aligns with the principles underlying the CDAI (28, 29). This underscores the potential of dietary antioxidants in preventing age-related conditions such as cardiovascular diseases (30), cancers (31) and neurodegenerative diseases. Essential dietary antioxidants, including *β*-carotene (32), vitamins C and E (33), selenium, zinc, manganese, and coenzyme Q10 (34), are crucial in countering the adverse effects of oxidative stress. Supplementation with antioxidants can help restore depleted endogenous antioxidant levels, thereby ultimately reducing oxidative damage.

Oxidative stress refers to the excessive production of highly active molecules in the body, such as reactive oxygen free radicals (ROS) and reactive nitrogen free radicals (RNS), the oxidation production exceeds the removal of oxides, resulting in tissue damage (33, 34). During brain aging, neurons and microglia accelerate apoptosis, mitochondrial dysfunction, release more ROS and RNS, metabolize into superoxides and hydroxyl radicals, induce increased activity of REDOX signaling cascade, resulting in impaired synaptic function, and further increase oxidative stress and oxidative damage (35). With the increase of age, the number of antioxidative enzymes decreases, which is a key component of the body’s antioxidative defense mechanism, and individuals’ adaptability to oxidative stress declines accordingly. The hippocampus, a critical brain region responsible for learning and memory, is particularly susceptible to oxidative stress. Due to the high metabolic activity and sensitivity of hippocampal neurons to oxidative damage, excessive ROS and RNS tend to accumulate in this area. Oxidative stress in the hippocampus leads to mitochondrial dysfunction, DNA damage, and protein oxidation, ultimately impairing neuronal survival and synaptic plasticity.

Antioxidant food sources are vital for patients with cognitive impairments, particularly in protecting the hippocampus. Dietary antioxidants reduce oxidative stress through bioactive molecules, regulate gene expression, and modulate cellular signaling pathways. For example, selenium protects hippocampal neurons by activating glutathione peroxidase and thioredoxin reductase, preventing lipid peroxidation, and maintaining neuronal membrane integrity. Zinc is an essential component of zinc-dependent antioxidant enzymes in the hippocampus and is crucial for glutamatergic synaptic transmission and synaptic stability (36). The metabolite of vitamin A, retinoic acid (RA), supports hippocampal synaptic plasticity and alleviates age-related cognitive decline. Further analysis of the correlation between CDAI components and CERAD (memory) revealed that carotenoids and vitamin E contribute the most to the antioxidant effects of CDAI. Non-enzymatic antioxidant vitamin E effectively reduces oxidative stress (8), while carotenoids exhibit potent antioxidant properties by neutralizing reactive oxygen species (ROS) and regulating inflammation, thereby significantly protecting hippocampal neurons. Thus, dietary intake of antioxidant-rich nutrients holds promise in preventing and mitigating cognitive impairment caused by oxidative stress. However, the precise molecular mechanisms warrant further investigation.

Balanced nutrient intake contributes to overall health during the aging process, and excessive intake of single nutrients is detrimental to both cognitive function and health. Moreover, the intake of single antioxidants cannot counteract the oxidative stress under pathological conditions and might even interfere with the body’s redox balance. Therefore, the CDAI index was introduced in this study. The CDAI serves as a quantitative measure to assess the aggregate antioxidant capacity associated with specific dietary intake, utilized across diverse research investigations. Evidence suggests that increased CDAI scores are associated with a lowered susceptibility to age-related diseases such as cardiovascular disease and cancers (37, 38). Our study provides additional evidence that CDAI scores are associated with lower cognition. Regardless of other significant covariates, this association remains consistent and meaningful. Higher CDAI scores showed a protective effect in cognitive assessments, suggesting that CDAI scores play a prominent role in immediate and delayed learning abilities in the elderly when acquiring new linguistic information. Specific mechanisms require further investigation. For clinicians, CDAI can help identify deficiencies in antioxidant intake, thereby guiding personalized dietary interventions focused on improving CDAI scores and promoting cognitive performance. It can also be used to track changes in diet quality and evaluate the effectiveness of dietary adjustments. However, the composite nature of CDAI may make direct dietary recommendations more challenging. To address this, clinicians can prioritize recommending antioxidant-rich, nutrient-dense foods and supplements to help individuals optimize their dietary structure and health outcomes. In public health, priority should focus on high-risk populations, such as older adults and those with low antioxidant intake. Early screening tools are essential to identify individuals at risk of cognitive impairment, enabling targeted dietary education and continuous monitoring. CDAI offers a practical and evidence-based approach for assessing dietary antioxidant intake and evaluating interventions, particularly at the regional level. Public health initiatives can use CDAI to design targeted campaigns promoting antioxidant-rich foods. Incorporating a lifelong cognitive health framework into national dietary guidelines is crucial. This approach promotes antioxidant-rich dietary patterns early in life to prevent cognitive decline, addressing the challenges of an aging population.

Nomograms provide an effective way to communicate multifactorial predictions in a straightforward and accessible manner. In this study, the nomogram model could be used to predict the risk of cognitive impairment and even dementia, which will help clinicians to better identify high-risk groups of cognitive impairment. Adding more predictors could improve the nomogram’s performance, we have incorporated additional variables, such as physical activity, depression and comorbidities, into the model and reassessed its performance. Considering the model’s sensitivity and the AUC, it is more suitable as a supplementary tool rather than a standalone diagnostic method. Integrating the model into a multi-modal diagnostic framework is also an option to enhance its practical utility. Certainly, future studies are necessary to validate the model in external populations to enhance its generalizability and ensure its clinical applicability.

To enhance the nomogram model’s utility and accuracy, future research should integrate biomarkers like oxidative stress and neuroinflammatory markers to strengthen its biological foundation and insights into cognitive decline. Longitudinal data validation is crucial to confirm its predictive stability across diverse populations. Additionally, machine learning techniques can improve the model by analyzing complex datasets, identifying novel predictors, and dynamically adapting to different cohorts.

This study demonstrated an association between CDAI scores and cognitive function within a nationally representative sample of individuals over 60 years in the United States. Although similar studies have investigated the relationship between dietary antioxidants and cognitive outcomes (39), our study offers several important distinctions. First, the CDAI in our study was constructed using a different combination of antioxidant nutrients, including carotenoids instead of manganese. Second, we emphasized domain-specific cognitive outcomes and found a particularly strong association between CDAI and memory performance, which is crucial for early dementia detection. Most importantly, we developed and validated a nomogram prediction model, enhancing the clinical applicability of our findings. These contributions, built upon shared data sources but employing distinct variable definitions, analytical approaches, and clinical perspectives, underscore the novelty and added value of our research.

However, certain limitations must be acknowledged. First, this study was a cross-sectional analysis, we cannot determine the causal relationship between CDAI scores and cognitive performance. Second, the 24-h dietary recall interview is questionnaire-based, so dietary assessments may involve measurement error and inaccuracies. Lastly, we excluded participants with missing information, and we could not determine whether these individuals affected the results. We emphasize the need for prospective cohort studies to track dietary interventions and their impact on cognitive changes over time. Furthermore, randomized controlled trials are essential to assess the effects of antioxidant supplementation or specific dietary strategies on cognitive decline.

## 5 Conclusion

In conclusion, there is a significant negative association between CDAI and low cognition in the U.S. elderly population, with higher CDAI scores having a protective effect on cognitive function. A nomogram was constructed to predict the risk of cognitive impairment, which will help clinicians to better identify high-risk groups of cognitive impairment. This study provides a new way to explore dietary intervention for cognitive function impairment.

## Data Availability

Publicly available datasets were analyzed in this study. This data can be found at: the original data that support the findings of this study are openly available in NHANES (https://www.cdc.gov/nchs/nhanes).
