# Supplementary material for: Association between composite dietary antioxidant index and cognitive function impairment in the elderly: evidence from NHANES 2011–2014
Source: Front Neurol. 2025 Apr 25;16:1529989. doi: 10.3389/fneur.2025.1529989 (PMC12061890; doi:10.3389/fneur.2025.1529989)
Supplement: Supplementary file 1 [file Table_1.docx]

Supplementary Material

# Supplementary Tables

| **Table S1. The missing data rates of covariates in our dataset.** | |
| --- | --- |
| **Variable** | **Missing (%)** |
| Physical activity | 877 (34.75) |
| PIR | 191 (7.57) |
| CKD | 110 (4.36) |
| BMI | 32 (1.27) |
| Alcohol | 18 (0.71) |
| Depression | 16 (0.63) |
| Stroke | 5 (0.20) |
| Marital status | 3 (0.12) |
| Educational attainment | 2 (0.08) |
| Smoke | 2 (0.08) |

**Table S2. The multiple logistic regression analysis to explore the associations between CADI score and cognitive performance.**

| **Variables** | **CERAD** | | **AFT** | | **DSST** | |
| --- | --- | --- | --- | --- | --- | --- |
|  | **OR (95% CI)** | ***P*** | **OR (95% CI)** | ***P*** | **OR (95% CI)** | ***P*** |
| **CDAI** | 0.94(0.90,0.98) | 0.014 | 0.98(0.92,1.04) | 0.389 | 0.98(0.92,1.04) | 0.462 |
| **Age** | 1.10(1.06,1.14) | <0.001 | 1.07(1.05,1.10) | <0.001 | 1.13(1.09,1.18) | <0.001 |
| **Sex** |  |  |  |  |  |  |
| Female | 1.00 (reference) | 1.00 (reference) | 1.00 (reference) | 1.00 (reference) | 1.00 (reference) | 1.00 (reference) |
| Male | 2.48(1.55,3.95) | 0.002 | 1.44(0.88,2.33) | 0.125 | 1.98(1.52,2.58) | <0.001 |
| **Race** |  |  |  |  |  |  |
| Mexican American | 1.00 (reference) | 1.00 (reference) | 1.00 (reference) | 1.00 (reference) | 1.00 (reference) | 1.00 (reference) |
| Non-Hispanic Black | 0.60(0.36,1.01) | 0.053 | 2.16(1.20,3.91) | 0.016 | 1.19(0.74,1.94) | 0.429 |
| Non-Hispanic White | 0.56(0.32,0.95) | 0.035 | 0.71(0.44,1.14) | 0.134 | 0.19(0.12,0.30) | <0.001 |
| Other Hispanic | 1.34(0.79,2.27) | 0.237 | 1.50(0.82,2.72) | 0.163 | 1.79(0.95,3.37) | 0.068 |
| Other Race - Including Multi-Racial | 0.53(0.28,1.01) | 0.053 | 2.10(1.17,3.79) | 0.019 | 0.22(0.08,0.61) | 0.008 |
| **Educational attainment** |  |  |  |  |  |  |
| High school and above | 1.00 (reference) | 1.00 (reference) | 1.00 (reference) | 1.00 (reference) | 1.00 (reference) | 1.00 (reference) |
| Less than high school | 2.05(1.30,3.23) | 0.006 | 1.49(0.99,2.25) | 0.057 | 3.60(2.38,5.45) | <0.001 |
| **Marital status** |  |  |  |  |  |  |
| Divorced | 1.00 (reference) | 1.00 (reference) | 1.00 (reference) | 1.00 (reference) | 1.00 (reference) | 1.00 (reference) |
| Married | 1.02(0.61,1.72) | 0.918 | 1.16(0.62,2.18) | 0.595 | 0.92(0.45,1.89) | 0.807 |
| Other * | 1.19(0.63,2.25) | 0.556 | 0.99(0.56,1.75) | 0.959 | 1.03(0.51,2.08) | 0.930 |
| Separated | 0.63(0.25,1.59) | 0.287 | 0.74(0.28,1.97) | 0.506 | 1.18(0.28,4.91) | 0.802 |
| **BMI** | 0.98(0.94,1.02) | 0.220 | 1.01(0.96,1.05) | 0.728 | 1.00(0.95,1.04) | 0.863 |
| **PIR** | 0.86(0.76,0.96) | 0.014 | 0.84(0.75,0.93) | 0.003 | 0.71(0.60,0.84) | 0.001 |
| **Smoke** |  |  |  |  |  |  |
| No | 1.00 (reference) | 1.00 (reference) | 1.00 (reference) | 1.00 (reference) | 1.00 (reference) | 1.00 (reference) |
| Yes | 0.76(0.56,1.04) | 0.083 | 0.92(0.67,1.26) | 0.553 | 0.92(0.58,1.47) | 0.709 |
| **Alcohol** |  |  |  |  |  |  |
| No | 1.00 (reference) | 1.00 (reference) | 1.00 (reference) | 1.00 (reference) | 1.00 (reference) | 1.00 (reference) |
| Yes | 0.85(0.59,1.22) | 0.336 | 0.71(0.50,0.99) | 0.046 | 0.72(0.44,1.17) | 0.158 |
| **Day1 intake energy** | 1.00(1.00,1.00) | 0.168 | 1.00(1.00,1.00) | 0.446 | 1.00(1.00,1.00) | 0.437 |
| **Day2 intake energy** | 1.00(1.00,1.00) | 0.190 | 1.00(1.00,1.00) | 0.304 | 1.00(1.00,1.00) | 0.052 |
| **Stroke** |  |  |  |  |  |  |
| No | 1.00 (reference) | 1.00 (reference) | 1.00 (reference) | 1.00 (reference) | 1.00 (reference) | 1.00 (reference) |
| Yes | 0.99(0.54,1.82) | 0.970 | 1.27(0.73,2.19) | 0.351 | 2.56(1.33,4.94) | 0.010 |
| **CKD** |  |  |  |  |  |  |
| No | 1.00 (reference) | 1.00 (reference) | 1.00 (reference) | 1.00 (reference) | 1.00 (reference) | 1.00 (reference) |
| Yes | 1.38(1.01,1.88) | 0.042 | 1.15(0.83,1.58) | 0.360 | 1.80(1.17,2.78) | 0.013 |
| **Diabetes** |  |  |  |  |  |  |
| No | 1.00 (reference) | 1.00 (reference) | 1.00 (reference) | 1.00 (reference) | 1.00 (reference) | 1.00 (reference) |
| Yes | 0.89(0.66,1.18) | 0.374 | 0.90(0.64,1.28) | 0.528 | 1.24(0.85,1.80) | 0.233 |
| **Hypertension** |  |  |  |  |  |  |
| No | 1.00 (reference) | 1.00 (reference) | 1.00 (reference) | 1.00 (reference) | 1.00 (reference) | 1.00 (reference) |
| Yes | 1.57(1.08,2.30) | 0.024 | 1.41(0.96,2.06) | 0.073 | 1.37(0.83,2.28) | 0.190 |
| **Depression** |  |  |  |  |  |  |
| No | 1.00 (reference) | 1.00 (reference) | 1.00 (reference) | 1.00 (reference) | 1.00 (reference) | 1.00 (reference) |
| Yes | 1.25(0.70,2.22) | 0.405 | 1.91(0.83,4.40) | 0.115 | 2.06(1.01,4.17) | 0.047 |
| **Physical activity** |  |  |  |  |  |  |
| No | 1.00 (reference) | 1.00 (reference) | 1.00 (reference) | 1.00 (reference) | 1.00 (reference) | 1.00 (reference) |
| Yes | 1.09(0.77,1.54) | 0.591 | 0.88(0.66,1.18) | 0.353 | 1.84(1.31,2.60) | 0.003 |
| Note: low cognitive performance was defined by CERAD total scores ≤21; low cognitive performance was defined by AFT total scores ≤13; low cognitive performance was defined by DSST total scores ≤33; *, represents widowed, never married and living with partner.Abbreviations: PIR, Poverty-income ratio; BMI, Body mass index; CERAD, Consortium to Establish a Registry for Alzheimer’s Disease; AFT, Animal Fluency Test; DSST, Digit Symbol Substitution Test. | | | | | | |

**
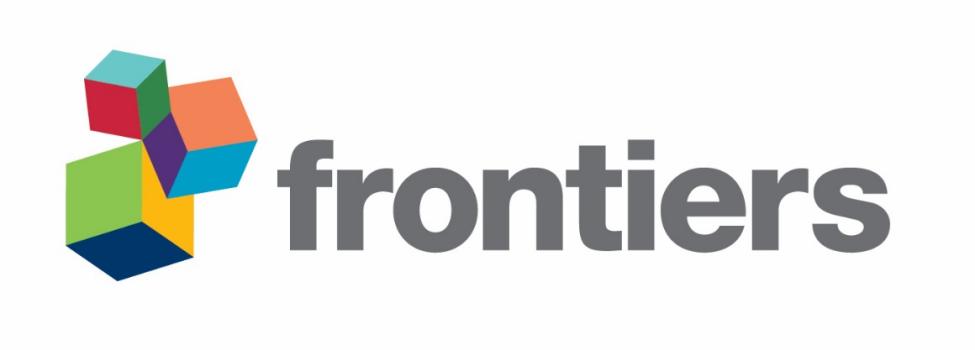
**
